# Preliminary Experience of Liquid Biopsy in Lung Cancer Compared to Conventional Assessment: Light and Shadows

**DOI:** 10.3390/jpm12111896

**Published:** 2022-11-12

**Authors:** Marco Montella, Giovanni Ciani, Vincenza Granata, Roberta Fusco, Francesca Grassi, Andrea Ronchi, Immacolata Cozzolino, Renato Franco, Federica Zito Marino, Fabrizio Urraro, Riccardo Monti, Roberto Sirica, Giovanni Savarese, Ugo Chianese, Angela Nebbioso, Lucia Altucci, Maria Teresa Vietri, Valerio Nardone, Alfonso Reginelli, Roberta Grassi

**Affiliations:** 1 Pathology Unit, Department of Mental and Physical Health and Preventive Medicine, University of Campania “Luigi Vanvitelli”, 80138 Naples, Italy; 2 Department of Precision Medicine, Università degli Studi della Campania “Luigi Vanvitelli”, 80138 Naples, Italy; 3 Division of Radiology, Istituto Nazionale Tumori IRCCS Fondazione Pascale-IRCCS di Napoli, 80131 Naples, Italy; 4 Medical Oncology Division, Igea SpA, 80013 Napoli, Italy; 5 AMES-Centro Polidiagnostico Strumentale, SRL, 80013 Naples, Italy; 6 Italian Society of Medical and Interventional Radiology (SIRM), SIRM Foundation, 20122 Milan, Italy

**Keywords:** liquid biopsy, conventional biopsy, lung cancer

## Abstract

Purpose: To assess the qualitative relationship between liquid biopsy and conventional tissue biopsy. As a secondary target, we evaluated the relationship between the liquid biopsy results and the T stage, N stage, M stage, and compared to grading. Methods: The Local Ethics Committee of the “Università degli Studi della Campania Luigi Vanvitelli”, with the internal resolution number 24997/2020 of 12.11.2020, approved this spontaneous prospective study. According to the approved protocol, patients with lung cancer who underwent Fine-Needle Aspiration Cytology (FNAC), CT-guided biopsy, and liquid biopsy were enrolled. A Yates chi-square test was employed to analyze differences in percentage values of categorical variables. A *p*-value < 0.05 was considered statistically significant. Data analysis was performed using the Matlab Statistic Toolbox (The MathWorks, Inc., Natick, MA, USA). Results: When a genetic mutation is present on the pathological examination, this was also detected on the liquid biopsy. ROS1 and PDL1 mutations were found in 2/29 patients, while EGFR Exon 21 was identified in a single patient. At liquid biopsy, 26 mutations were identified in the analyzed samples. The mutations with the highest prevalence rate in the study populations were: ALK (Ile1461Val), found in 28/29 patients (96.6%), EML4 (Lys398Arg), identified in 16/29 (55.2%) patients, ALK (Asp1529Glu), found in 14/29 (48.3%) patients, EGFR (Arg521Lys), found in 12/29 (41.4%) patients, ROS (Lys2228Gln), identified in 11/29 (37.9%) patients, ROS (Arg167Gln) and ROS (Ser2229Cys), identified in 10/29 (34.5%) patients, ALK (Lys1491Arg) and PIK3CA (Ile391Met), identified in 8/29 (27.6%) patients, ROS (Thr145Pro), identified in 6/29 (20.7%) patients, and ROS (Ser1109Leu), identified in 4/29 (13.8%) patients. No statistically significant differences can be observed in the mutation rate between the adenocarcinoma population and the squamous carcinoma population (*p* > 0.05, Yates chi-square test). Conclusions: We showed that, when a genetic mutation was detected in pathological examination, this was always detected by liquid biopsy, demonstrating a very high concordance rate of genomic testing between tissues and their corresponding mutations obtained by liquid biopsy, without cases of false-negative results. In addition, in our study, liquid biopsy highlighted 26 mutations, with the prevalence of ALK mutation in 96.6% of patients, supporting the idea that this approach could be an effective tool in cases with insufficient tumor tissue specimens or in cases where tissue specimens are not obtainable.

## 1. Introduction

The assessment of tumor genetic alterations is currently crucial in oncological patient management and treatment decisions [1,2,3,4,5,6,7,8,9,10]. In fact, in the era of personalized medicine, an accurate genetic tumor assessment allows to identify the more precise treatment, to define the treatment time, and to standardize the follow-up time [11,12,13,14,15,16,17,18,19,20,21]. Usually, evaluation of tumor mutational status is performed by means of a section of the primary or secondary lesion [22,23,24,25]. However, this approach shows several weaknesses, such as the invasive nature of the procedure, the inaccessibility when the lesion is in a deep site, and the risk of an inadequate sample or a partial assessment, considering intra-tumor heterogeneity, especially spatial heterogeneity, when a single biopsy is tested [26]. Moreover, this approach does not allow a dynamic follow-up of cancer molecular modifications that could occur during treatment [26]. In addition, in oncological patients, a critical point is related to the possibility of an early cancer diagnosis. It is known that patients have a higher cure rate and five-year survival if diagnosed at early stages [2,27,28,29,30,31]. To perform large-scale tumor screening among healthy people and to obtain an accurate mutational cancer status, several researchers and companies have turned their attention toward liquid biopsy [31,32,33,34,35,36,37]. Blood includes several categories of biological substances, such as circulating cells, extracellular vesicles, platelets, protein, mRNA, miRNA, and cell-free DNA (cfDNA) [36]. In oncological patients, a cfDNA portion, known as circulating tumor DNA (ctDNA), is liberated by lesions due to active release, necrosis, and apoptosis [37]. The alterations in ctDNA can be considered as a biomarker, and these can allow to identify cancer patients from a group of healthy individuals, to evaluate the cancer mutational status, to assess the response to treatment, and to identify groups of patients with a poor prognosis. This approach could manage the highest level of personalized medicine [38,39,40,41,42,43,44,45,46,47,48,49]. Compared to conventional tissue biopsy, liquid biopsy is more feasible and less invasive and is more comprehensive to assess tumor heterogeneity since all tumor parts release ctDNA [38].

Worldwide, lung cancer is one of the leading causes of morbidity and mortality among oncological patients [50,51,52,53]. Despite the progress in the treatment, also thanks to the benefits from immunotherapy, there is a necessity to identify new robust biomarkers that could predict response, resistance, and/or toxicity to treatment [54,55,56,57]. Up to now, the European Medicines Agency (EMA) [58] and the FDA [59] have approved epidermal growth factor receptor (EGFR) mutation testing using ctDNA in non-small-cell lung cancer (NSCLC) patients. For patients treated with immune checkpoint inhibitors for NSCLC, several studies showed that ctDNA could be an early marker of therapeutic efficacy and it could better predict survival outcomes [60].

ctDNA is highly fragmented, ranging from 100 to 10,000 bp. It is challenging to isolate ctDNA from the blood for quantitation since the small fragments are easy to lose or degrade [26]. Although the concentration of ctDNA should increase with the stage and tumor size, the total percentage of ctDNA in the blood is extremely low, putting many requirements on the sample processing procedure [26]. Thus, ctDNA assays used for early cancer diagnosis should be highly sensitive. However, highly sensitive assays are always expensive, making large-scale practical applications unrealistic. For late-stage cancer tumor typing, the sensitivity can be moderate because the concentration of ctDNA is much larger [26]. Additionally, it has been shown that both the concentration and stability of ctDNA could be influenced by the form, release, degradation, and clearance of cfDNA [26]. Until now, very few studies have discussed the clearance rate and biological mechanism of ctDNA. Another significant obstacle at present is the lack of biological knowledge and experimental evidence to support the quantitative relationship between ctDNA and early cancer development [26].

Another critical issue is the lack of biological knowledge and experimental evidence to support the qualitative and quantitative relationship between ctDNA and conventional pathological tissue.

This research is a part of an ongoing study on liquid biopsy in lung cancer. We assessed as a primary endpoint the qualitative relationship between ctDNA and conventional cytological sampling biopsy. As a secondary target, we evaluated the relationship between the liquid biopsy results and the T stage, N stage, M stage, and grading.

## 2. Methods

### 2.1. Patient Selection

The Local Ethics Committee of the “Università degli Studi della Campania Luigi Vanvitelli”, with internal resolution No. 24997/2020 of 12.11.2020, approved this prospective study.

According to the approved protocol, patients with histologically confirmed lung cancer who underwent tissue CT-guided biopsy and liquid biopsy were enrolled.

The study was performed in accordance with relevant guidelines and regulations.

### 2.2. Subjects’ Selection

The eligibility of the patients has been assessed by the investigators. Inclusion criteria included: (a) age ≥ 18 years, (b) suitable mental health conditions, (c) ability to sign a specific informed consent form, (d) diagnosis of NSCLC with histological confirmation, (e) computed tomography staging, and (f) blood sample to perform liquid biopsy.

Exclusion criteria included: (a) age less than 18 years, (b) pregnancy, (c) absolute contraindication to CT study (previous adverse reactions to contrast medium), (d) inability to sign a specific informed consent form, (e) refusal to provide a blood sample for liquid biopsy, and (f) absence of conventional biopsy.

Having to compare the proportions of genetic mutations between two groups, we used the following statistical inference model: inference for two proportions. To obtain a statistical power of 80% and a statistical significance of 0.1, and an error of the second type of 0.2, starting from a difference in incidence between the proportions of genetic mutations between the two groups of 10%, it will be necessary to include at least 116 cases (58 per group).

In this study, we report the preliminary results of the first 29 enrolled patients.

## 3. Sampling Protocol

The study included patients with a newly detected pulmonary nodule, who had been scheduled to undergo transthoracic CT-guided Fine-Needle Aspiration Cytology (FNAC) and a final cytological diagnosis of NSCLC. The patients were thoroughly informed of risks, complications, and the possible inadequacy of the rate of the FNAC procedures and gave their written informed consent to undergo the procedure.

A 23-G, 150 mm needle connected to a syringe mounted on the holder was used in all cases.

Thereafter, a radiologist, with the collaboration of a cytopathologist, performed a CT-guided percutaneous transthoracic FNAC using a Revolution Discovery 64-slice CT scanner (General Electric, Boston, MA, USA). ROSE (Rapid On-Site Evaluation) was always performed to provide indications on the number of passes and the choice of vials in CT-guided cases. According to our protocol [13], a total of 1 to 4 passes were performed for each patient. A total of 1 to 2 smears were air-dried and DiffQuik-stained for ROSE, and additional smears were alcohol-fixed and Papanicolaou-stained. Residual material in the hub of the needle from the first pass, as well as material from the additional passes, was suspended in formalin for cell block (CB) preparation.

All the patients were clinically monitored in the recovery room, and 2 h after the procedure, a chest radiography was performed to diagnose possible complications.

## 4. Morphophenotypical Assessment

Cytological diagnoses were matched with the corresponding histological diagnoses, when available, and all diagnoses were conducted according to the last WHO classification of tumors criteria (WHO 2021). All cytological and histological slides were evaluated by two experienced pathologists with specific expertise in cytology.

Immunocytochemistry (ICC) was carried out on CB sections for diagnostic purposes to classify pulmonary non-small-cell carcinomas. ICC was performed on the Ventana platform (VENTANA BenchMark Ultra system; Ventana, Tucson, Arizona) and included the following antibodies: TTF1 (thyroid transcription factor 1), p63, Napsin, p40, cytokeratin 7 (CK7), PD-L1 (programmed death ligand-1), ALK (anaplastic lymphoma kinase), and ROS1. The ICC markers used were TTF1, Napsin, and CK7 for adenocarcinoma, and p63, p40, and CK 5/6 for squamous cell carcinoma.

## 5. Molecular and Predictive Tests

The smears were used for the extraction of DNA for next-generation sequencing, whereas CB sections were used for ICC (ALK, ROS1, and PD-L1). Next-generation sequencing was performed to analyze epidermal growth factor receptor (EGFR) and BRAF mutational status in all cases of lung adenocarcinoma. In all cases, smears were used for the extraction of DNA by the Qiamp DNA Micro Kit (Qiagen, Hilden, Germany), according to the manufacturer’s instructions. Extracted DNA was eluted in 20 μL of elution buffer and subsequently quantified by a Qubit 2.0 Fluorometer (Life Technologies, Carlsbad, CA, USA) using the Qubit dsDNA HS Assay kit, according to the manufacturer’s recommendations. The extracted DNA was stored at −20 °C.

## 6. Liquid Biopsy Analysis

- Blood Samples and ctDNA Extraction

Here, 5 mL of blood was collected by ethylenediaminetetraacetic acid (EDTA) blood collection tubes, the same day as CT-guided Fine-Needle Aspiration Cytology.

To remove blood cells, blood was centrifuged at 1800× *g* for 10 min at 4 °C. Then, the supernatant was centrifuged at 16,000× *g* for 10 min at 4 °C to remove any remaining cells. Circulating tumor DNA was extracted from 2 mL of plasma, by digestion in 100 μL of proteinase K buffer for 10 min at 37 °C, followed by purification with the Plasma XS kit with the given protocol. The purified ctDNA was quantified by a Picogreen fluorescence assay using the provided lambada DNA standards.

- ctDNA Sequencing and Analysis

The 5-biotinylated probe solution was provided as a capture probe, and the baits target cancer-related genes. Hybridization, target amplification, barcode library preparation, and size selection were performed according to the manufacturer’s protocols. Libraries were prepared with the TruSigt Oncology 500 ctDNA kit, based on target enrichment that analyzes 523 cancer-relative genes. The assay detected several classes of genetic mutations, such as small nucleotide variants (SNVs), indels, splice variants, and biomarkers such as tumor mutational burden (TMB) and microsatellite instability (MSI). Sequencing was performed on an Illumina NovaSeq 6000 (San Diego, CA, USA) platform. The analysis was performed with the TruSight Oncology local app on a Dragen server.

### Statistical Analysis

A Yates chi-square test was employed to analyze differences in percentage values of categorical variables.

A *p*-value < 0.05 was considered statistically significant.

Data analysis was performed using the MATLAB Statistical Toolbox (The MathWorks, Inc., Natick, MA, USA).

## 7. Results

We assessed 29 patients with NSCLC. In 21/29 patients, the lesion was adenocarcinoma, while 8/29 patients had squamous carcinoma. Tumor lesions were localized in the peripheral area in 86.2% (25/29 patients), while 4/29 (13.8%) lesions were localized in the central area.

**Figure 1:**
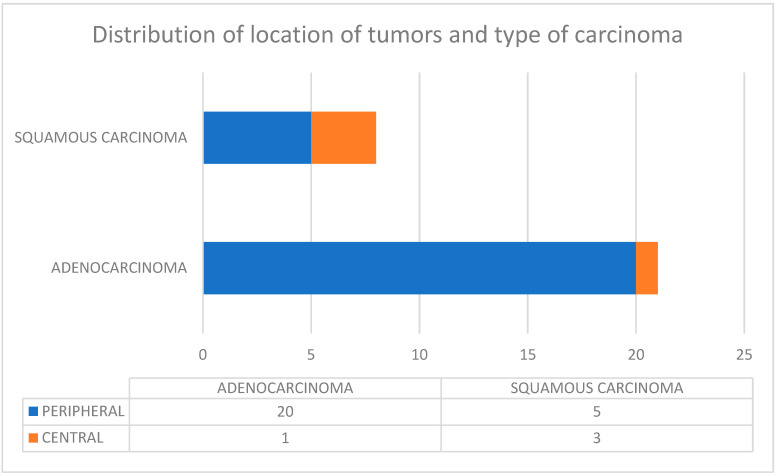
Distribution of location of tumors and type of carcinoma.

Figure 1 describes the distribution of the location of tumors and the type of carcinoma.

Table 1 reports the TNM and stage distribution in our population.

All the genetic mutations, detected by pathological analysis, were found by liquid biopsy. There were no false negatives at liquid biopsy compared to pathological analysis.

Table 2 reports the mutations found at pathological analysis: ROS1 and PDL1 mutations were found in 2/29 (at immunocytochemical analysis) patients, while EGFR Exon 21 was identified in a single patient (at RT-PCR analysis). Mutations were observed in the adenocarcinoma group.

**Figure 2:**
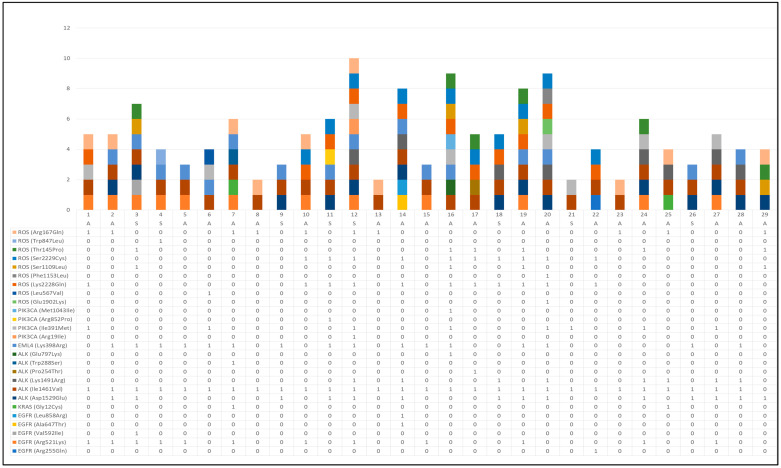
Mutation status at liquid biopsy for a single patient (A = adenocarcinoma and S = squamous carcinoma).

At liquid biopsy, 26 mutations were identified in the analyzed samples. Figure 2 reports the mutation status at liquid biopsy for a single patient.

The mutations with the highest prevalence rate in the study populations were:

- ALK (Ile1461Val), found in 28/29 (96.6%) patients;
- EML4 (Lys398Arg), identified in 16/29 (55.2%) patients;
- ALK (Asp1529Glu), found in 14/29 (48.3%) patients;
- EGFR (Arg521Lys), found in 12/29 (41.4%) patients;
- ROS (Lys2228Gln), identified in 11/29 (37.9%) patients;
- ROS (Arg167Gln) and ROS (Ser2229Cys), identified in 10/29 (34.5%) patients;
- ALK (Lys1491Arg) and PIK3CA (Ile391Met), identified in 8/29 (27.6%) patients;
- ROS (Thr145Pro), identified in 6/29 (20.7%) patients;
- ROS (Ser1109Leu), identified in 4/29 (13.8%) patients;
- KRAS (Gly12Cys) was found in 2/29 (6.9%) patients; and
- EGFR (Arg255Gln), EGFR (Val592Ile), EGFR (Ala647Thr), EGFR (Leu858Arg), ALK (Pro254Thr), ALK (Trp288Ser), ALK (Glu797Lys), PIK3CA (Arg19Ile), PIK3CA (Arg852Pro), PIK3CA (Met1043Ile), ROS (Glu1902Lys), ROS (Leu567Val), ROS (Phe1153Leu), and ROS (Trp847Leu) were identified only in one patient.

Table 3 reports the mutation status at liquid biopsy compared, respectively, to T, N, M, and grading.

No statistically significant differences were observed among the mutation status and the disease status (*p* > 0.05, Yates chi-square test).

**Figure 3:**
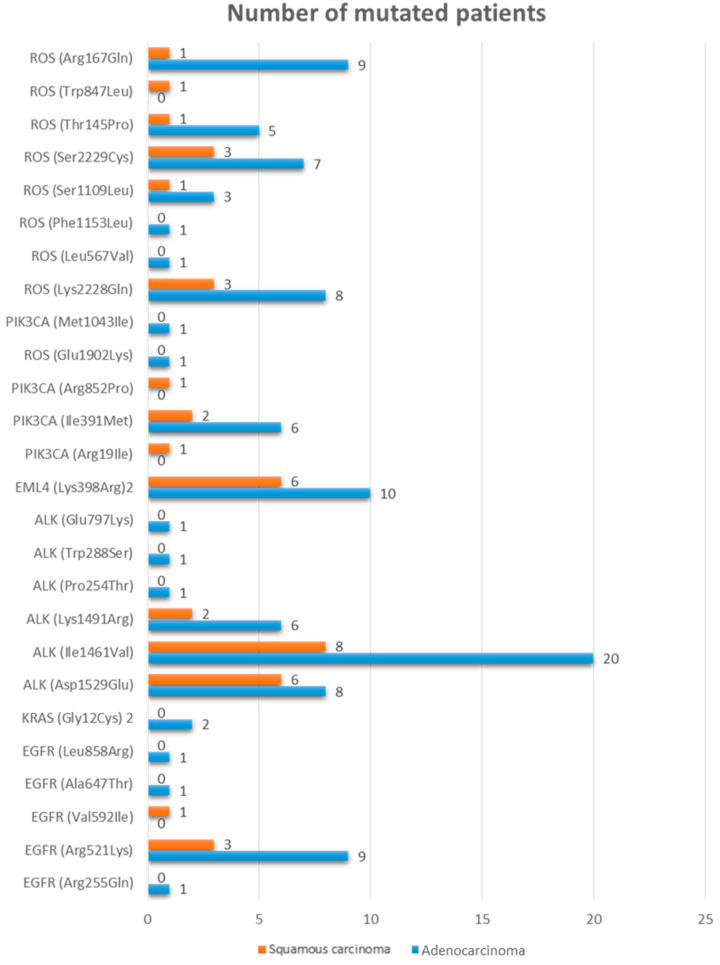
Number of mutated patients in the adenocarcinoma and squamous carcinoma groups.

Figure 3 reports the number of mutated patients compared between the adenocarcinoma population and the squamous carcinoma population. No statistically significant differences were observed in the number of each mutation between the two populations (*p* > 0.05, Yates chi-square test, Table 3).

EGFR (Arg255Gln), EGFR (Ala647Thr), EGFR (Leu858Arg), KRAS (Gly12Cys), ALK (Pro254Thr), ALK (Trp288Ser), ALK (Glu797Lys), ROS (Glu1902Lys), PIK3CA (Met1043Ile), ROS (Leu567Val), and ROS (Phe1153Leu) mutations were present only in patients with adenocarcinoma.

In the adenocarcinoma group, the main mutations were ALK (Ile1461Val), in 95.24% of patients, EML4 (Lys398Arg) in 47.62% of the patients, and EGFR (Arg521Lys) and ROS (Arg167Gln) in 42.86% of the patients (Table 3).

EGFR (Val592Ile), PIK3CA (Arg19Ile), PIK3CA (Arg852Pro), and ROS (Trp847Leu) mutations were present only in the squamous carcinoma population.

In the squamous carcinoma group, the main mutations were ALK (Ile1461Val), in 100% of patients, and EML4 (Lys398Arg) in 75% of the patients (Table 4).

**Figure 4:**
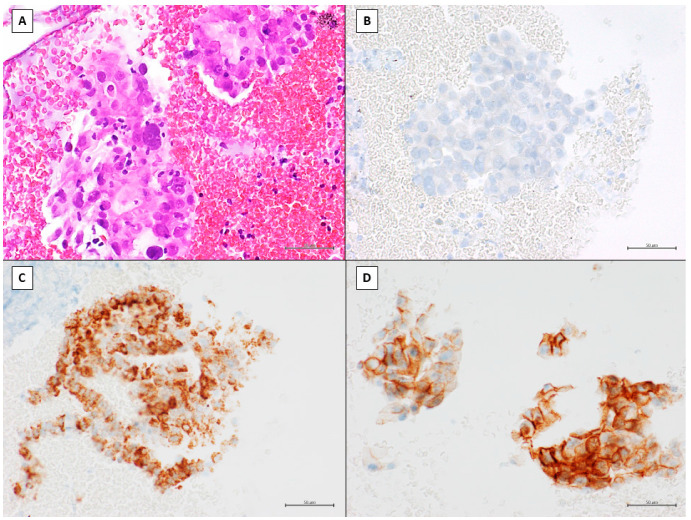
(**A**) CB section of NSCLC adenocarcinoma (40× magnification, scale bar 50 µm, H/E staining). (**B**–**D**) Immunocytochemistry evaluation: (**B**) ALK-negative (Clone D5F3), (**C**) ROS1-positive (Clone SP384), and (**D**) PDL1-positive & gt; 50% (Clone SP263) (ICC assay on Ventana Benchmark XT, 40× magnification, scale bar 50 µm).

Figure 4 shows the CB section of NSCLC adenocarcinoma and the immunocytochemistry evaluation.

## 8. Discussion

At present, there are two significant settings in which the liquid biopsy could provide an improvement to NSCLC patients: at the first molecular diagnosis and at the progression during targeted therapy [61]. A precise assessment of genetic mutations, at both baseline and progression, is critical for patient management, and to understand the process that causes therapies’ resistance [62]. Although repetitive tissue biopsies may represent the tools to monitor the tumor evolution, the feasibility of these procedures is not applicable for all patients. Therefore, several liquid biopsy platforms have been established as a complementary tool to conventional tissue biopsy and as a feasible means of identifying acquired resistance mechanisms [63,64,65,66,67,68]. Today, liquid biopsy is proposed in the new College of American Pathologists (CAP)/International Association for the Study of Lung Cancer (IASLC)/Association for Molecular Pathology (AMP) guideline for molecular testing of patients with NSCLC [69]. This approach cannot be a substitute of conventional tissue biopsy, but instead an alternative in cases with insufficient tumor tissue specimens or when a tissue specimen is not accessible [61]. In addition, circulating biomarkers are hypothetically more likely to reveal tumor burden and are more useful in representing cancer heterogeneity. Despite these benefits, there are several issues regarding sensitivity and utility, so due to its function in clinical settings, these should be evaluated in supplementary studies.

In this ongoing prospective study, at the time of writing, we assessed 29 patients with NSCLC: 21/29 with adenocarcinoma and 8/29 with squamous carcinoma. Tumor lesions were localized in the peripheral area in 86.2% (25/29 patients) and in 13.8% (4/29) in the central area. According to our results, all the genetic mutations detected in pathological analysis were found by liquid biopsy. There were no false negatives at liquid biopsy compared to pathological analysis. Although the great reliability of the results obtained in cytology has already been proven [70,71], we are well-aware that molecular analysis on cytological samples is not without limitations, as the tumor cell quality, quantity, and purity can greatly vary from specimen to specimen [72]. Despite this, cytological specimens are the main samples used for the diagnosis of advanced lung cancer and expression rates in cytological samples are not statistically different from histological samples [73].

The mutations with the highest prevalence rate in the study populations were: ALK (Ile1461Val), found in 28/29 patients (96.6%), EML4 (Lys398Arg), identified in 16/29 (55.2%) patients, ALK (Asp1529Glu), found in 14/29 (48.3%) patients, EGFR (Arg521Lys), found in 12/29 (41.4%) patients, ROS (Lys2228Gln), identified in 11/29 (37.9%) patients, ROS (Arg167Gln) and ROS (Ser2229Cys), identified in 10/29 (34.5%) patients, ALK (Lys1491Arg) and PIK3CA (Ile391Met), identified in 8/29 (27.6%) patients, ROS (Thr145Pro), identified in 6/29 (20.7%) patients, and ROS (Ser1109Leu), identified in 4/29 (13.8%) patients. KRAS (Gly12Cys) was found in 2/29 (6.9%) patients, and EGFR (Arg255Gln), EGFR (Val592Ile), EGFR (Ala647Thr), EGFR (Leu858Arg), ALK (Pro254Thr), ALK (Trp288Ser), ALK (Glu797Lys), PIK3CA (Arg19Ile), PIK3CA (Arg852Pro), PIK3CA (Met1043Ile), ROS (Glu1902Lys), ROS (Leu567Val), ROS (Phe1153Leu), and ROS (Trp847Leu) were identified only in one patient.

These results are similar to the data obtained by Kong et al. [74]. In addition, in our study, liquid biopsy was able to highlight 26 mutations, with the prevalence of the ALK (Ile1461Val) mutation in 96.6% of patients. These data disagree with several studies on conventional biopsy, which confirmed the presence of ALK fusion genes in 2–7% of NSCLC, arising more commonly in nonsmokers and almost exclusively in tumors of non-squamous histology, although this result was obtained by liquid biopsy [56]. Regarding the adenocarcinoma group, the main mutations were ALK (Ile1461Val) in 95.24% of patients, EML4 (Lys398Arg) in 47.62% of the patients, and EGFR (Arg521Lys) and ROS (Arg167Gln) in 42.86% of the patients. In the squamous carcinoma group, the main mutations were ALK (Ile1461Val) in 100% of patients and EML4 (Lys398Arg) in 75% of the patients. The EGFR (Val592Ile), PIK3CA (Arg19Ile), PIK3CA (Arg852Pro), and ROS (Trp847Leu) mutations were assessed only in patients with squamous carcinoma. Our results suggested that liquid biopsy has the ability to offer much of the data needed for clinical application of targeted therapy. The discovery of oncogenic driver mutations of the EGFR gene and the authorization of EGFR inhibitors have modified the therapy in NSCLC [75]. Patients with EGFR exon 19 deletions or exon 21 L858R mutations are suitable for treatment with afatinib, gefitinib, dacomitinib, erlotinib, and osimertin [69]. Most of the FDA-approved EGFR mutation detection tests are based on conventional biopsies. However, several plasma-based assays have been introduced for providing a non-invasive tool for patients not suitable for tissue biopsy. Liquid biopsy allows the clinicians to assess therapy effectiveness and disease progression over time. Liu et al. [75] assessed the blood samples of 24 NSCLC patients and 6 age-matched healthy donors, comparing the EGFR mutation profile detected from CTCs and cfDNA to matched tumor tissues. They showed that the results from this non-invasive EGFR mutation analysis were encouraging, and this combined workflow could represent a valuable means for informing therapy selection and for monitoring treatment of patients with NSCLC [75].

We found no statistically significant differences among the mutation status and the disease status (*p* > 0.05, Yates chi-square test) and no statistically significant differences could be observed in the number of each mutation between patients with adenocarcinoma and patients with squamous carcinoma (*p* > 0.05, Yates chi-square test), probably due to the small sample size of the populations. Unlike our results, Van der Linden et al. showed that by using a variant allele frequency threshold of 1%, somatic variants were detected in 23.5% of patients, with a median variant allele fraction of 3.65%. By using this threshold, they could discriminate early-stage lung cancer patients from the control group [76].

The detection of cfDNA offers prospects for screening, diagnosis, treatment assessment, and disease surveillance. Increasing evidence has highlighted the clinical utility of detecting mutations in cfDNA, and the amount of cfDNA in circulation has been correlated with the tumor burden [77]. The liquid biopsy offers advantages to the treatment-naive patient by saving tissue for additional assessment, comprising immunohistochemistry. In addition, this approach is simple and less expensive, providing intra-tumor heterogeneity and high sensitivity for detecting tumor burden [77]. However, not all tumors release adequate quantities of DNA. Treatment-naive patients with indolent, slow-growing tumors could be at higher risk of false-negative results in plasma compared to patients with a more disseminated cancer [75].

This study has several limits. Firstly, the small sample size makes accurate comparisons problematic, and therefore the analysis should be performed with a larger number of patients to verify and to validate the findings, and this represents a future end-point of our study. Secondly, the analysis was focused on the first diagnosis, so we had no data on genetic mutations during progression. Finally, the meanings of the 26 mutations that we found and what role these might play in patient management were not discussed and should be explored in future studies.

## 9. Conclusions

In this ongoing prospective study, we assessed 29 patients with NSCLC: 21/29 with adenocarcinoma and 8/29 with squamous carcinoma. All the genetic mutations detected by pathological analysis were found by liquid biopsy. There were no false negatives at liquid biopsy compared to pathological analysis. In addition, in our study, liquid biopsy was able to highlight 26 mutations, with the prevalence of the ALK mutation in 96.6% of patients, supporting the idea that this approach could be an effective tool in cases with insufficient tumor tissue specimens or in cases where tissue specimens are not obtainable. Additionally, circulating biomarkers are more likely to reveal tumor burden and are more useful in representing cancer heterogeneity. Despite these advantages, there are a number of issues concerning sensitivity and utility and the role of liquid biopsy in clinical settings that require further investigations.

## Figures and Tables

**Table 1:** TNM and stage distribution.

Value	T (No. Patients)	N (No. Patients)	M (No. Patients)	Stage (No. Patients)
0		20	21	
1	15	1	8	11
2	7	5		4
3	4	3		6
4	3			8

**Table 2:** Mutations found in pathological analysis.

Mutation	Number of Mutations Found in Adenocarcinoma Patients
EGFR Exon 21	1
ROS1	2
ALK	0
PDL1	2

**Table 3:** Mutation status at liquid biopsy compared, respectively, to T, N, and M, and to grading.

		EGFR	KRAS (Gly12Cys)2	ALK	EML4 (Lys398Arg)2	PIK3CA	ROS
**Mutation Status Compared to T Stage**							
Negative	1	7	14	0	8	10	3
	2	3	6	0	2	6	1
	3	3	4	0	3	3	2
	4	2	3	0	0	1	1
Positive	1	8	1	15	7	5	12
	2	4	1	7	5	1	6
	3	1	0	4	1	1	2
	4	1	0	3	3	2	2
*p*-Value, Yates’ Chi-Square		0.97	0.83	1.0	0.60	0.92	0.91
**Mutation status compared to N stage**							
Negative	0	11	18	0	9	15	5
	1	0	1	0	1	0	0
	2	1	5	0	2	4	0
	3	3	3	0	1	1	2
Positive	0	9	2	20	11	5	15
	1	1	0	1	0	1	1
	2	4	0	5	3	2	5
	3	0	0	3	2	2	1
*p*-Value, Yates’ Chi-Square		0.54	0.33	1.0	0.99	0.94	0.57
**Mutation status compared to M stage**							
Negative	0	10	20	0	10	16	4
	1	5	7	0	3	4	3
Positive	0	11	1	21	11	5	17
	1	3	1	8	5	4	5
*p*-Value, Yates’ Chi-Square		0.76	0.93	1.0	0.94	0.36	0.58
**Mutation status compared to grading**							
Negative	1	5	11	0	4	9	3
	2	2	3	0	4	3	0
	3	3	6	0	2	4	1
	4	5	7	0	3	4	3
Positive	1	6	0	11	7	2	8
	2	2	1	4	0	1	4
	3	3	0	6	4	2	5
	4	3	1	8	5	4	5
*p*-Value, Yates’ Chi-Square		0.94	0.91	1.0	0.60	0.77	0.91

**Table 4:** Mutation status in adenocarcinoma and squamous carcinoma groups.

Mutation	Adenocarcinoma	%	Squamous Carcinoma	%	*p*-Value, Yates’ Chi-Square Test
EGFR (Arg255Gln)	1	4.76	0	0	0.61
EGFR (Arg521Lys)	9	42.86	3	37.5	0.93
EGFR (Val592Ile)	0	0.00	1	12.5	0.61
EGFR (Ala647Thr)	1	4.76	0	0	0.61
EGFR (Leu858Arg)	1	4.76	0	0	0.61
KRAS (Gly12Cys) 2	2	9.52	0	0	0.93
ALK (Asp1529Glu)	8	38.10	6	75	0.17
ALK (Ile1461Val)	20	95.24	8	100	0.66
ALK (Lys1491Arg)	6	28.57	2	25	0.79
ALK (Pro254Thr)	1	4.76	0	0	0.61
ALK (Trp288Ser)	1	4.76	0	0	0.61
ALK (Glu797Lys)	1	4.76	0	0	0.61
EML4 (Lys398Arg)2	10	47.62	6	75	0.36
PIK3CA (Arg19Ile)	0	0.00	1	12.5	0.61
PIK3CA (Ile391Met)	6	28.57	2	25	0.79
PIK3CA (Arg852Pro)	0	0.00	1	12.5	0.61
PIK3CA (Met1043Ile)	1	4.76	0	0	0.61
ROS (Glu1902Lys)	1	4.76	0	0	0.61
ROS (Lys2228Gln)	8	38.10	3	37.5	0.69
ROS (Leu567Val)	1	4.76	0	0	0.61
ROS (Phe1153Leu)	1	4.76	0	0	0.61
ROS (Ser1109Leu)	3	14.29	1	12.5	0.63
ROS (Ser2229Cys)	7	33.33	3	37.5	0.82
ROS (Thr145Pro)	5	23.81	1	12.5	0.87
ROS (Trp847Leu)	0	0.00	1	12.5	0.61
ROS (Arg167Gln)	9	42.86	1	12.5	0.27

## Data Availability

All data are reported in this manuscript.
